# Examining priorities and investments made through the Global Financing Facility for maternal and newborn health: a sub-analysis on quality

**DOI:** 10.1080/16549716.2024.2406486

**Published:** 2024-10-11

**Authors:** Meghan Bruce Kumar, Mary Kinney, Joël Kiendrébéogo, Donat Shamba, Joy E. Lawn, Peter Waiswa

**Affiliations:** a Department of Nursing, Midwifery and Health, Northumbria University, Newcastle upon Tyne, UK; b Department of Health System and Research Ethics, KEMRI-Wellcome Trust Programme, Nairobi, Kenya; c School of Public Health, University of the Western Cape, Cape Town, South Africa; d Department of Research, Expertise and Capacity Building, Recherche pour la santé et le développement (RESADE), Ouagadougou, Burkina Faso; e Department of Public Health, University Joseph Ki-Zerbo, Ouagadougou, Burkina Faso; f Heidelberg Institute of Global Health, Medical Faculty and University Hospital, Heidelberg University, Heidelberg, Germany; g Institute of Tropical Medicine, Department of Public Health, Antwerp, Belgium; h Department of Health Systems, Impact Evaluation and Policy, Ifakara Health Institute, Dar es Salaam, Tanzania; i School of Public Health, Makerere University College of Health Sciences, Kampala, Uganda; j London School of Hygiene & Tropical Medicine, Department of Infectious Disease Epidemiology and International Health, London, UK

**Keywords:** Global Financing Facility for Women, Children and Adolescents: Examining National Priorities, Processes and Investments, Maternal and newborn health, quality of care, Global Financing Facility, health systems strengthening, content analysis

## Abstract

Improving quality of care could avert most of the 4.5 million maternal and neonatal deaths and stillbirths that occur each year. The Global Financing Facility (GFF) aims to catalyse the national scale-up of maternal and newborn health (MNH) interventions through focused investments. Achieving impact and value for money requires high, equitable coverage and high quality of interventions. This study examines whether the rhetoric of increasing coverage together with quality has informed investment strategies in MNH through a secondary analysis of 25 GFF documents from 11 African countries. The analysis shows that the country GFF-related documents incorporate some MNH-related quality of care components; however, there is a lack of clarity in what is meant by quality and the absence of core MNH quality of care components as identified by the World Health Organization’s MNH quality framework, especially experience of care and newborn care. Many of the Investment Cases have a more diagonal focus on MNH service delivery considering the clinical dimensions of quality, while the investments described in the Project Appraisal Documents are primarily on horizontal structural aspects of the health system strengthening environment. The GFF is at the forefront of investing in MNH globally and provides an important opportunity to explicitly link health systems investments and quality interventions within the MNH continuum of care for optimal impact.

## Paper context

- **Main findings:** The analysis of maternal and newborn health-related quality of care in Global Financing Facility country documents reveals variability in depth and content, with most documents focusing on horizontal approaches to health system strengthening and if specific to maternal and newborn health, the focus is on provision of quality maternal care with little to no attention on experience of care and quality newborn care.
- **Added knowledge:** This content analysis is the first to examine quality and maternal and newborn health within the content of Global Financing Facility documents showing that patient experience, stillbirths, and specific quality newborn indicators are seldom mentioned.
- **Global health impact for policy and action:** The analysis underscores the need for more diagonal approaches to address the highest impact interventions for maternal and newborn health and the need to focus on person-centered care within the Global Financing Facility related processes for the investment to have maximum impact.

## Background

Trends for maternal and newborn mortality and stillbirths have stagnated or slowed in the past decade, even though the majority of the world’s births now occur in facilities (83%) [1]. As such, the global discourse on maternal and newborn health (MNH) has shifted from increasing access to health services to increasing ‘effective’ coverage of health services, which encompasses both coverage and quality of care as critical for achieving impact [2,3]. The World Health Organization (WHO) has clearly defined their vision for MNH care as ‘every pregnant woman and newborn receives quality care throughout pregnancy, childbirth and the postnatal period’, a vision operationalised through two main pillars – provision of care and experience of care- in a quality framework linked to a monitoring framework and recommended indicators [4–6]. Although quality is typically measured in specific health areas with focused indicators (a ‘vertical’ approach), it is enabled by ‘horizontal’ health system strengthening across areas like human resources, information systems, financing and other building blocks [7]. This linkage between horizontal investment to achieve health area-focused gains is termed a ‘diagonal’ approach [8]. MNH can be viewed as a vertical area where measurable improvements in quality require both horizontal and vertical investments [9,10].

It is unknown whether the rhetoric of increasing coverage together with quality has informed investment strategies in MNH. One vehicle for investment in this health area, the Global Financing Facility (GFF), was set up as a catalytic funding mechanism to ‘ensure all women, children and adolescents can survive and thrive’ [11]. GFF-related investments are described in two country documents: investment cases (ICs), designed to describe the need for investment in reproductive, maternal, newborn, child, adolescent health (RMNCAH) in a country; and the project appraisal documents (PADs), which describe the GFF’s grant financing along with other co-financing by the World Bank in the form of credits, loans and sometimes other donors [12].

## Secondary analysis approach

This paper presents findings related to quality of care as a secondary analysis of a published content analysis that examined MNH in 25 GFF-related policy documents from 11 African countries between 2015–2019 [13]. Supplementary file 1 presents search terms and more detail of methods applied, including a structured content analysis that incorporated a set of both broad quality terms and MNH-specific terms [14]. Country selection, data extraction and analysis can be found in the primary study [13]. For quality in MNH, we applied the same M^3^ framework to qualitatively examine content that is further described in that paper. Specifically, by assessing documents in three areas: broad intention and framing (Mindset, M1), as well as detailed indicators (Measures, M2), and linked funding (Money, M3), the analysis brought together content analysis and qualitative thematic analysis around priority setting with linked quantitative data on specific interventions and earmarked funding amounts [12]. A summary statement about quality and MNH was developed for each GFF document and each component (mindset, measures, money) and then a scoring system was applied to grade the extent of quality MNH inclusion. (Supplementary file 2). We note that although the term ‘mentions’ is used at times in the text of this article, the full analytical approach involved looking in depth at the context and depth of each occurrence of a given concept and related terms.

## Reflections on ‘quality’ in the GFF documents

**Figure 1.**
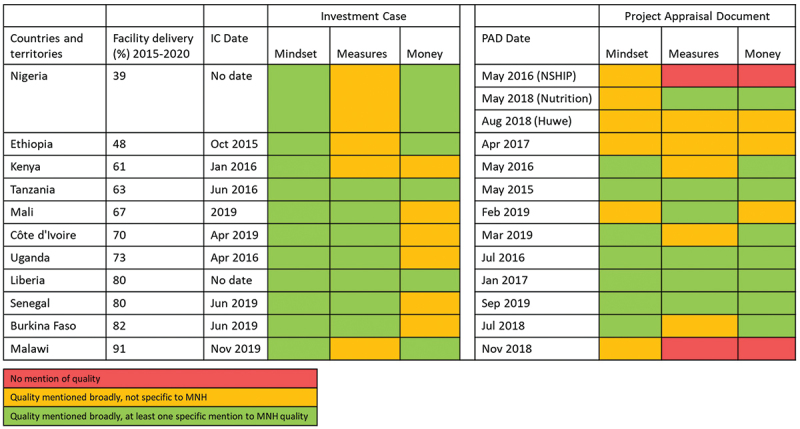
Extent to which quality and MNH content are included in documents.

Quality-related content specific to MNH is mentioned in most of the GFF documents including in the funding descriptions, as shown in Figure 1. The ICs all have content on MNH-related quality for mindset, but some documents do not include specific measures and money even though they include quality more broadly. The PADs have more variability. The two PADs that did not include anything on quality were focused on nutrition and early childhood development. The actual content, in terms of depth and focus, varies by country and document reflecting the range of approaches applied to strengthening health systems in different contexts, perhaps making the common horizontal approaches easier to describe [15]. We showcase two strong country examples to give an idea of how this works in practice (Boxes 1 and 2).

**Box 1:** 

COUNTRY HIGHLIGHT - Liberia GFF documents and quality MNH In Liberia, health systems building blocks were mentioned throughout the IC and linked directly to MNH items. Two of the six priority investment of the IC relate directly to MNH-related quality including EmONC and maternal neonatal death surveillance and response (MNDSR). MNH interventions are included in other priority areas, such as community engagement with direct linkage to quality efforts: ‘The MNDSR process will be triggered and led by community members hearing about a death in their area (page 47)’. These priorities are reflected in the measures and money components. The IC includes the comprehensive indicator list for the broader National Investment Plan for a Resilient Health System, 2016-2021 which includes both broad quality and specific MNH quality indicators e.g. maternal neonatal death surveillance and response, human resources (i.e. midwife skills, retention, and numbers), community (family-oriented services, ANC/PNC) and commodities (stock outs specific to MH). The results framework for the RMNCAH program also includes two MNH-related quality specific indicators: proportion of hospitals with 100% CEmONC compliance and basic equipment availability for BEmONC. The description of the investment package reflects the priorities outlined in the investment case, specifically linked to MNH-related quality interventions, i.e. EmONC, MNDSR, and strengthen referral systems, as well as broader quality and health systems strengthening, i.e. quality assurance and leadership and governance. Itemized costs are also provided for MNDSR and EmONC interventions including health workforce, infrastructure, data and information systems, medical supplies and diagnostics, and service delivery. This is both a good example of how this can be done as well as a potential opportunity to learn from the process of developing this technically robust IC. This level of clarity is less present in the PAD for Liberia.

**Box 2:** 

COUNTRY HIGHLIGHT – Uganda’s project appraisal document In Uganda, quality of care is mentioned throughout the PAD and specifically noted as weak around the time of birth (EmONC) and for referrals. Project components on strengthening health systems include sections on quality, and quality is specifically funded ($8.5 m). Where health systems strengthening is funded, MNH is mentioned in the project component descriptions of these funds, especially MDPSR, EmONC, referrals, midwifery care.

In general, quality is mentioned frequently across the documents; although there was a lack of clarity in what is meant by quality or ‘high-quality’, with few definitions provided in the documents. The exception is the Ethiopia’s IC, which provides a clear definition and also mentions improving ‘*patient safety, effectiveness and patient-centredness’*, with a plan for a new national strategy on quality to be developed. In general, quality is implied to mean something akin to access or coverage. For example, Tanzania’s IC stated: ‘*Improve quality of care at all levels of service delivery and health administration through health system strengthening and capacity development to achieve high population coverage of high impact RMNCAH interventions including nutrition in an integrated manner*’. A few country documents place quality more at the facility-level, focusing on accreditation (eg Ethiopia PAD: ‘*Quality of services will be a measure to be obtained from the health facility surveys*’).

Specific to MNH, we assessed how many documents mentioned (at least once) core concepts related to quality MNH (Table 1). Emergency Obstetric and Newborn Care (EmONC), midwifery and referral were included in nearly all of the ICs and many of the PADs. Within documents, EmONC was frequently mentioned (*n* = 19/25) and often described as part of the project components for health system strengthening, focused in improving technical quality of care. Overall, midwives (*n* = 19) and referral (*n* = 21) were less of a conceptual focus although mentioned in similar numbers of documents, highlighting opportunities to strengthen human resource management and continuity of care. Maternal and perinatal death surveillance and response (MPDSR), which can inform quality improvement processes, is explicitly mentioned in seven ICs and five PADs (*n* = 12). The mentions relating to MPDSR ranged from a core focus in both documents (e.g. Liberia) to specific sections dedicated to the intervention process (e.g. Ethiopia IC) to only one mention (e.g. Burkina Faso PAD). By type of document, ICs had more focus on the service delivery areas than PADs and focused primarily on technical quality.

**Table 1.** GFF policy documents with mentions of core concepts relating to quality MNH.

Core concepts relating to quality MNH*	% of documents with key quality concepts (# documents/total documents)
**Investment cases (*n* = 11)**	
EmONC	100% (11/11)
Midwives	91% (10/11)
Referral	100% (11/11)
MPDSR	64% (7/11)
Family Centred Care	9% (1/11)
Respectful maternity care	64% (7/11)
**Project appraisal documents (*n* = 14)**	
EmONC	57% (8/14)
Midwives	64% (9/14)
Referral	71% (10/14)
MPDSR	36% (5/14)
Family Centred Care	7% (1/14)
Respectful maternity care	0% (0/14)

*Search terms related to these concepts can be found in supplementary file 1; results by country and by document can be found in supplementary file 2.

Patient experience related to MNH, such as respectful maternity care or family-centered care, is almost never mentioned even though it is the second of two pillars of the WHO MNH quality of care framework [4]. Two documents (Cote d’Ivoire IC and Kenya PAD) mentioned family centredness, each only once; seven ICs included content on respectful care linked to MNH but no PADs mentioned it. Stillbirths, argued to be a sensitive marker of the quality of MNH care [16], are rarely mentioned in the GFF documents and not at all in relation to quality of care [13], a missed opportunity to show impact linking to coverage and quality of maternal healthcare.

In the ICs and PADs, health systems investments are clearly linked to quality in theory with key MNH coverage indicators (four antenatal visits or skilled delivery) along with broader quality-related indicators. Yet, these linkages are not always clear. Specifically related to measures, EmONC related indicators are in seven documents and MPDSR-related indicators are in three documents. Generally, quality indicators in PADs were more structural or horizontal and less clinical or technical, focused on aspects like facility accreditation and availability of services rather than key interventions. The global measurement roadmap for MNH includes quality-related indicators, many of which are not included at all in the GFF documents [2], especially for newborn-related quality interventions. The recommended MNH quality indicators in the roadmap might be a starting point for future GFF work, although even here patient experience is not well-represented nor is it clear whether countries will willingly adopt them. Concepts of experience of care and its measurement are relatively new on the global agenda, especially for newborn care, and indicators need to be defined and routinised.

## Reflections on quality and ‘horizontal’ health systems strengthening investment against ‘vertical’ MNH priorities

GFF is focusing on a ‘vertical’ or specific health area (RMNCAH, within which the MNH population comprising the greatest burden of deaths), but generally investing as part of a funding consortium led by the World Bank that is investing horizontally in health system strengthening. Therefore, we expected to see a clear connection between MNH and health system investments in a diagonal approach to financing through targeted MNH-related quality interventions, such as human resources especially midwives, strengthening referral systems, MPDSR and EmONC. Yet in many documents (both ICs and PADs), health system interventions and investments were primarily described horizontally (eg financial management, procurement/supply chain, information systems). Horizontal approaches to addressing quality are an important first step, and more MNH-targeted investments are needed to address the highest burden areas. Most documents had at least one MNH-specific quality component in the measures and money, such as strengthening midwifery (Kenya) or MPDSR (Uganda) (See Supplementary File 2 for more examples). In reality, there is no ‘magic bullet’ or single intervention to improve MNH quality of care and multiple approaches are needed, including multi-level, multi-component interventions that are dynamic, context-specific, and adaptive [15]. This is reflected in the GFF documents assessed.

By type of document, the ICs – which are country-led and GFF-supported – have more diagonal focus on system strengthening for MNH service delivery than PADs – which are linked to World Bank projects, and focus more on horizontal approaches, indicators and investments. To some extent, this might be expected in PADs, as they do not describe total MNH financing, where the government or other donors might input to overcome financing gaps [12,17]. In Box 1, we show a positive exemplar of the Liberian IC that had frequent, explicit linkages between health systems investment and MNH care and impact. Box 2 presents the Uganda PAD as a positive example linking MNH and quality indicators.

## Final reflections

This secondary analysis of the GFF-related country documents in 11 African countries shows that MNH-related quality of care content while present, varies across country GFF-related documents. The lack of consistency between countries and across documents (ICs or PADs) within countries made content analysis challenging, especially on quality, which has multiple approaches. We were limited to the documents reviewed, and acknowledge quality of care may be the focus of other country and GFF-related documents. Nonetheless, the approach we applied enabled us to identify some common patterns, including inconsistent content or gaps [18]. As with the primary study [13], we found that most of the quality-related MNH content focused on maternal health interventions with little content on quality newborn care, even when expanding our search to newborn specific interventions identified as important to track for quality (e.g. Kangaroo Mother Care) [2]. Additionally, the absence or limited content related to experience of care (respectful care, family centredness) presents an opportunity for the GFF in country engagements to broaden the existing focus on structural and clinically-driven aspects of quality to improve and increase the focus on person-centredness, continuity of and experience of respectful, family-centred care. The GFF has prioritized quality in their most recent strategic plan [19], and future accountability efforts could assess how the quality components may have changed in their more recent documents. In all settings, a focus on multi-dimensional quality covering structural, technical and person-centredness aspects along all stages of the maternal and newborn care is critical for ending preventable maternal and newborn deaths and stillbirths and reducing related morbidities.

## Supplementary Material

Supplemental Material

## Data Availability

The datasets used and/or analysed in this study are available from the corresponding author on reasonable request.
