# Supplementary material for: Examining priorities and investments made through the Global Financing Facility for maternal and newborn health: a sub-analysis on quality
Source: Glob Health Action. 2024 Oct 11;17(1):2406486. doi: 10.1080/16549716.2024.2406486 (PMC11486310; doi:10.1080/16549716.2024.2406486)
Supplement: Supplemental Material [file ZGHA_A_2406486_SM1338.docx]

SUPPLEMENTARY FILES

# Supplementary file 1: Data extraction related to MNH quality

## Data extraction components related to quality

| **Category** | **Search terms** |
| --- | --- |
| Quality | - Quality - Quality of care - Quality assurance |
| MNH continuum of care quality constructs | - midw* - referral - "family" - looking for family-centred - "respect" - looking for respectful care/disrespect & abuse - MPDSR (used “MPDSR”, “MDSR”, “audit”, “death review”) |
| MNH search terms related to quality to identify if specific interventions relate to quality. See full list of MNH related search terms in original paper [13] | - EmONC (used “EmOC”, “obstetric” to identify) - Resus (neonatal resuscitation) - Perinatal - Preterm, prem - Kangaroo, KMC - “birth weight” - Small and sick newborn - Neonatal infection - “sepsis” – include only for newborn or neonatal “sepsis” |

## Data extraction process

First, we searched each document for the term “quality” to see if it was mentioned, how it was defined, and which dimensions and measures of quality were included. For technical dimensions of MNH relating to quality, we considered specific terms including referral and comprehensive obstetric care, midwife/wives and audit to identify content relating to Maternal and Perinatal Death Surveillance and Response (MPDSR). We also explored how health systems were portrayed and whether this was linked to quality and MNH interventions, considering WHO health system building blocks [1], concepts of service delivery, private sector, Civil and Vital Registration System (CVRS), universal health coverage (UHC) as possible areas where health system interventions might impact quality. The inclusion of family-centred care and respect allowed for the assessment of representation of patient experience dimensions of quality. Once terms were identified in the documents, we used a standard extraction tool to make high-level observations around how quality was included broadly as well as specific to MNH.

## GFF MNH analysis data extraction tool

***Instructions:***

- *Save file as: Country name/ IC or PAD / date of extraction” [DDMMYYYY]*
- *Complete data extraction by answering questions in each section and providing summary points or copying in text from document in bullet form to respond to the below questions*
  - *Include page numbers for content*
  - *Include screen shots of relevant tables*
- *To find the information, use the search terms in the excel file.*

Prepared by NAME

Date: DATE

Country: NAME

Document: INDICATE IC OR PAD

**Provide details about the document:**

- Title:
- Date of publication
- Total pages

**Respond to the questions or statements for each section. Use quotes and screen shots to verify information and include page numbers. Enter in summary for each point within numbered topic areas. Provide 3-5 bullet point summarizing information and main take aways.**

1. Newborn (search terms: Newborn, Neonat*, Stillb*, Perinatal)
   1. Definition and description
      1. Copy definition if available
   2. Count of total mentions (but not extract text) and mentions as part of acronym
   3. For each mention:
      1. Where is it in the document (ToC, Foreword, Situation Analysis, Indicators, Budget, etc)
         1. Is it consistently mentioned throughout the document, or does attention narrow or disappear as you move through to operational details, budgets, indicators? How so or why not?
         2. Any budget details at all?
      2. Is it mentioned mainly as part of a larger acronym/ as an ‘add on’ within other topics or are there independent sections/ detailed analyses and programmes specifically for newborns?
         1. If there are independent sections note what they are
   4. Framing: Is NB/SB integrated into maternal and/or child or brought out as a separate area for investment?
   5. Service delivery lens: what key interventions are included and how described, eg:
      1. Resuscitation
      2. preterm
      3. kangaroo
      4. low birth weight
      5. “small and sick”
      6. postnatal
      7. breast*

- *Add summary of main take aways here*

1. Maternal (search terms: matern*)
   1. Definition and description
      1. Copy definition if available
   2. Count of total mentions (but not extract text) and mentions as part of acronym
   3. For each mention:
      1. Where is it in the document (ToC, Foreword, Situation Analysis, Indicators, Budget, etc)
         1. Is it consistently mentioned throughout the document, or does attention narrow or disappear as you move through to operational details, budgets, indicators? How so or why not?
         2. Any budget details at all?
      2. Is it mentioned mainly as part of a larger acronym/ as an ‘add on’ within other topics or are there independent sections/ detailed analyses and programmes specifically for maternal?
         1. If there are independent sections note what they are
   4. Framing: How is mother-baby dyad or family-centred care mentioned?
   5. Service delivery lens: what key interventions are included and how described, eg:
      1. antenatal
      2. PMTCT
      3. skilled birth attend*
      4. obstetric / EmOC
      5. abortion

- *Add summary here in bullets*

1. Health systems: Enabling environment to effectively deliver services/interventions benefiting
   1. Framing: Provide high level observations of content (1-2 sentence). Which HSBB are mentioned explicitly in relation to MNH?
      1. Financing: budgeting and RBF
      2. Info/data: indicators and tracking progress
      3. Human resources
      4. Management/governance: MPDSR
      5. Community:
      6. Commodities:
      7. Service delivery – systems: referral, networks of care, quality
      8. Private sector
      9. CRVS
      10. UHC

*Add summary here in bullets*

1. Quality (search terms: quality, respectful care, referral, MPDSR)
   1. Framing: Do the GFF documents mention quality?
      1. If so, how do they define quality?
      2. If they define it, what are the different components of quality (e.g. technical quality, patient experience)
   2. (referral/networks): How are different levels and actors described? Are interactions described to provide high quality care? (provider-patient/family, provider-provider across levels of care, with communities)

*Add summary here in bullets*

1. What is the overall rationale of the document (IC or PAD)
   1. Is it holistic? Is it focused on one aspect e.g. nutrition specific
   2. How does it address inequity generally? How does it address vulnerable populations relating to MNH?
   3. Any other comments

*Add summary here in bullets*

## Data analysis

For quality MNH, we applied a framework to examine quality in terms of content (mindset), indicators (measures), and linked funding (money) [1-2]. A summary statement about quality and MNH was developed for each GFF document included for each component (mindset, measures, money) to further synthesize the results. The statements were drafted by two authors (MBK, MVK) drawing from the original data extraction from the primary analysis and then validated by checking the GFF document. A long summary was drafted for each component for quality and then separately for quality related to MNH. From there, a shorter summary was drafted and further synthesized. Then, using these summaries, we applied a scoring system to grade the extent of quality MNH inclusion by three levels:

| No mention of quality |
| --- |
| Quality mentioned broadly, not specific to MNH |
| Quality mentioned broadly, at least one specific mention to MNH quality |

References

1. Kinney MV, Kumar M, Kabore I, Kiendrébéogo J, Waiswa P, Lawn JE. Global Financing Facility investments for vulnerable populations: content analysis regarding maternal and newborn health and stillbirths in 11 African countries 2015 to 2019. Glob Health Action. 2024;17(1). doi: doi.org/10.1080/16549716.2024.2329369.

2. Collection: Global Financing Facility for Women, Children, And Adolescents: Examining national priorities, processes, and investments. Taylor & Francis Group: Global Health Action; 2024 [cited 2024 13 September]. Available from: https://www.tandfonline.com/journals/zgha20/collections/global-financing-facility-women-children.

# Supplementary file 2: Results table

**Table S2.1: High level summary of content relating to MNH quality in the GFF documents by country**

| **Country** | **Investment case** | **PAD** |
| --- | --- | --- |
| Burkina Faso | Mindset: Quality incorporated throughout particularly as an adjective “quality case management”. Focus on service delivery. EmOC, referral systems, midwives and respectful care, MDSR included.  Measure: Outcomes and outputs related to quality improvement. Specific to quality MNH includes EmONC service coverage.  Money: Investments for quality and MNH broadly; not specific to quality MNH | Mindset: Quality incorporated throughout as project focuses on performance based financing to improve quality of care for RMNCAH. EmOC, referral systems, midwives and respectful care, MDSR included.  Measures: Broad quality indicators eg QoC checklist (provision), exit interviews for services (experience), not specific quality MNH  Money: Quality is embedded as part of the project objectives. Specific to MNH, there is a funded sub-component on strengthening MNCAH with aspects related to quality obstetric and neonatal emergencies with specifics to EmONC commodities, equipment and capacity building, referral systems and strengthening maternal and perinatal death audit committees. |
| Cote d’Ivoire | Mindset: Quality mentioned broadly but not defined; primarily included as a background (poor quality) to explain excess mortality. EmOC, referral systems, midwives, MDSR included.  Measure: Quality indicators throughout results strategy with specific indicators for MNH quality (EmONC, c-section rate, maternal & newborn death reporting, # midwives)  Money: A budget line has been provided to “guarantee the quality of primary health care”, 62% of total budget; not specific to quality MNH | Mindset: Quality is a main objective of the programme; mentioned throughout. EmOC, referral systems, midwives, MDSR included.  Measure: Broad quality indicators but not specific to MNH e.g. average health facility quality score is core indicator; other linked indicators # health districts covered (PBF), number of people trained  Money: Quality is embedded as part of the project objectives. Specific to MNH, there is a funded sub-component on reproductive health and nutrition that includes establishing maternal and perinatal death review committees, strengthening referral systems, and strengthening EmONC. |
| Ethiopia | Mindset: Quality is a core component with clear definition. Content is broader than service delivery (e.g. leadership, regulation); EmOC target; content on referral systems, midwives, MDSR.  Measure: Targets and indicators for quality of health services, but not specific to quality MNH  Money: Budget presented by health system elements, quality components included; health services disaggregated by programme (including MNH) | Mindset: Core component and mentioned throughout often as an adjective to describe an action/activity. EmOC in background but not in actions; Nothing on quality of care for inpatient newborn care, referral or networks of care.  Measures: Broad quality indicators included. Specific to MNH, one DHI mentions quality: “improving *quality* of postnatal services by setting up Directorate”.  Money: Investments for quality broadly and specific to MNH (PNC directorate as DLI) |
| Kenya | Mindset: Quality has a dedicated section. Focused primarily on specific clinical aspects of quality. Content on EmONC, midwives, referral and MDSR  Measure: No quality MNH specific description; focus on utilization and quality related to reducing medicines and supplies stock outs and information system data quality.  Money: Describes links to PBF; no specifics for quality MNH. | Mindset: Mostly broad but does mention specifics about strengthening midwifery training; quality mindset focuses on clinical aspects only, including MPDSR. No content on EmONC or referral.  Measure: Broad approach incorporating health systems strengthening not specific to MNH; focus on activities over indicators.  Money: There is a funded sub-component on quality that includes primarily health systems indicators and improving midwifery specific to MNH |
| Liberia | Mindset: Quality incorporated throughout and prioritized for MNH but not defined. EmONC, referral systems, midwives and respectful care, MPDSR included.  Measure: Indicators specific to MNH quality: CEmONC compliance and BEmONC equipment  Money: Investment includes “Quality RMNCH service delivery” as part of EmONC ($7,4m) | Mindset: Quality is core objective and embedded throughout document, including project description but not defined. EmONC, referral systems, midwives and respectful care, MPDSR included.  Measure: Core indicator (new) “Health facility quality index score improvement at Target PBF hospitals and facilities” includes MNH aspects in the quality check list structure (Childbirth: Maternal-Newborn with main causes; Paediatric in patient care – Maternal Newborn Best Practice)  Money: Specific components of programme and linked investment include quality (support to quality service delivery; and human resources for health); within these components, referral and midwives are specifically mentioned for MNH including emergency newborn care. |
| Malawi | Mindset: Quality incorporated throughout broadly for RMNCAH but not defined. EmONC, referral systems, midwives, MPDSR included.  Measure: Many indicators broadly for quality; none specific to quality MNH    Money: Specific budget line for quality; specific inclusion of budget for MNH quality related interventions e.g. EMoNC, MDSR. | Mindset: Quality mentioned throughout but focus of project is on ECD; not defined.  Measure: None  Money: None |
| Mali | Mindset: Quality incorporated throughout broadly; not defined. EmONC, referral systems, midwives mentioned.  Measure: Broad quality indicators; indicators specific to MNH quality (c-section rate, % of CEmONC health facilities with a functional mini blood bank, basic equipment availability for BEmONC)  Money: Quality budgeted for more broadly, not specific to quality MNH but implied as part of RMNCAH | Mindset: Quality for MNHC services mentioned; no definition. Midwives and referral mentioned but not core components of document.  Measure: Broad quality indicators and specific to quality MNH (cause of death determined for maternal and child deaths included as project indicator)  Money: Funding description focuses on access and quality of a broad package (RMNCH+N) and related PBF but there is nothing specific on quality MNH. |
| Nigeria (PAD-NSHIP) | Mindset: Quality incorporated throughout for MNH but not defined or detailed. EmONC, referral and respectful care mentioned.  Measure: Primarily focused on coverage rather than quality but do use structural quality of care (health facility assessment scores) and accreditation in results framework. Not quality MNH specific.  Money: Priority investments are detailed, with expectation that they would come from domestic and aid financing. Includes MNH as a component of Basic Minimum Package of Health Services, with a list of services. | Mindset: Quality for MNHC services an overall objective; no definition. EmONC included. Midwives mentioned but not specific to midwifery strengthening.  Measure: Quality indicators included broadly; not specific to quality MNH  Money: Investments for quality broadly; not specific to quality MNH |
| Nigeria - HUWE | (no new IC; same as Nigeria NSHIP) | Mindset: Quality mentioned broadly – not specific to quality MNH; no definition  Measure: Quality indicator included broadly; not specific to quality MNH  Money: Investments for quality broadly; not specific to quality MNH |
| Nigeria – Nutrition | (no new IC; same as Nigeria NSHIP) | Mindset: Quality mentioned broadly – not specific to quality MNH; no definition  Measure: None  Money: None |
| Senegal | Mindset: Quality incorporated throughout broadly but not defined. EmOC, midwives and referral mentioned.  Measure: Quality indicators included broadly and specifically for MNH (neonatal mortality rate considered a marker on the quality of newborn care; quality of care score for antenatal care)  Money: Quality improvement is considered in different priorities of the IC with one specific budget line on improving quality of RMNCAH services, but not specific to MNH | Mindset: Quality mentioned as core component broadly, no definition. EmONC and midwives mentioned throughout.  Measure: Quality indicators included broadly and specifically for MNH (% of pregnant women having 4 antenatal care visits at standard quality, % births in health centres with functional EmONC base)  Money: Quality is a core aspect of the funding with specific sections linked to MNH under improving availability of RMNCAH-N services of adequate quality. It describes a strategy for mobile midwives and strengthening services for emergency obstetric care. |
| Tanzania | Mindset: Quality incorporated throughout broadly but not defined. EmONC, referral, respectful care and MPDSR mentioned.  Measure: Indicators specific to MNH quality (CEmONC, BEmOC)  Money: Investment for quality broadly included for RMNCAH interventions. Access and strengthening EmONC services and MNCH referrals are costed activities. | Mindset: Quality mentioned as core component and specifically to MNH, with focus on EmONC; no definition of quality. Referral mentioned.  Measure: Quality indicators included (e.g. scorecards) and specifically for MNCH services (CEmONC)  Money: Quality MNH incorporated into the funding descriptions as part of two disbursement lined indicators. The RBF scheme focuses on improving quality of MNCH at primary health care facilities, using availability of EmONC services as proxy measure. Balance Score Cards will also be used and include MNH components e.g. iron and folic acid supplementary for ANC attendees. |
| Uganda | Mindset: Quality incorporated throughout broadly, not defined. EmONC, MPDSR, midwives, referral, and MPDSR mentioned.  Measure: Indicators specific to MNH quality (% deliveries in EmONC Facilities in district; % Narrowing in midwives staffing (public + private) differences between districts and within districts)  Money: Quality considered but no separate budget line for quality or MNH. | Mindset: Quality mentioned as core component broadly and specific to MNH; no definition. EmONC, midwives, referral, and MPDSR mentioned.  Measure: Quality indicators included broadly and specifically for MNH (maternal deaths audited; health centre IVs offering caesarean sections)  Money: Investments for quality of care more broadly described linked to the RBF package and strengthening health systems with some elements specific to MNH incorporated including improving supplies (eg mama kits and vacuums), health workforce (eg midwives) and quality of care (eg maternal and perinatal audit). |
